# Periods and Content of Psychological Distress During Rotation-Based Training for Newly Graduated Nurses: A Six-Year Descriptive Study

**DOI:** 10.7759/cureus.98110

**Published:** 2025-11-29

**Authors:** Yukari Shimizu, Yoko Hamano

**Affiliations:** 1 Department of Nursing, Komatsu University, Komatsu, JPN; 2 Department of Nursing, Hayashi Hospital, Echizen, JPN

**Keywords:** clinical coach, interpersonal relationships, newly graduated nurses, rotation-based training, stress or psychological distress

## Abstract

Introduction: At the University of Fukui Hospital, a national university corporation in Japan, an original rotation-based training program is implemented for newly graduated nurses. In this educational program, nurses rotate through several hospital wards before being permanently assigned to one. Each rotation requires the development of new interpersonal relationships and the acquisition of new knowledge. This study aimed to clarify the periods and content of psychological distress experienced by newly graduated nurses during rotation-based training, and to identify the timing and nature of necessary support.

Methods: This quantitative descriptive study was conducted from April 2008 to December 2013. The participants were 299 newly graduated full-time nurses working at Fukui Hospital who provided informed consent, of whom 230 (76.9%) responded to the survey and were included in the analysis. Data were collected using a self-administered questionnaire that was developed for this study. Basic statistical analyses were performed using Microsoft Excel (Microsoft Corp., Redmond, WA). Free-text responses were analyzed using text-mining software to conduct word frequency and co-occurrence analyses. The study was approved by the Ethics Review Committee of the Nursing Department of Fukui University Hospital.

Results: The period identified as the most psychologically distressing was the first rotation in July, reported by 177 nurses (77.0%). This coincided with the third month of employment, which is commonly associated with heightened anxiety among new nurses. Some participants commented that although the rotation provided opportunities to learn various nursing skills, it also required starting new interpersonal relationships each time.

Conclusion: These findings suggest that support should be provided, particularly during the first rotation transition, to help newly graduated nurses build interpersonal relationships and adapt smoothly to new environments.

## Introduction

According to Japan’s Ministry of Health, Labour and Welfare [1], approximately 60,000 to 65,000 individuals take the national nursing licensure examination each year. Upon obtaining this qualification, nurses who have just graduated and started their first job (newly graduated nurses) begin working in hospitals or care facilities [2]. However, the percentage of these nurses who leave their positions within the first year of employment has remained at 7%-8%.

The Japanese Nursing Association conducted a survey in 2004 entitled “Survey on the Actual Conditions of Early Resignation Among Newly Graduated Nursing Staff” [3], which found that 8.8% of new nursing staff resigned within their first year [2]. This has slightly improved in recent years, with the resignation rate of newly graduated nurses being 8.8% in 2024 and 10.2% in 2022 [2].

One of the factors contributing to the difficulty in retaining newly graduated nursing staff is the gap between the competencies acquired through basic nursing education and the skills required in actual clinical settings [4]. According to Uchino and Shimada, who conducted a literature review on the resignation of newly graduated nurses in Japan, the main factors associated with early turnover include reality shock and interpersonal relationships in the workplace [4]. Furthermore, psychological and physical health problems are common reasons for resignation among newly graduated nurses. The emotional immaturity and vulnerability often observed in today’s younger generation have also been cited. The sense of stagnation experienced by newly graduated nurses in their first year was primarily due to a lack of knowledge and underdeveloped technical skills [4,5].

At Fukui University Hospital, a comprehensive nursing competency development program incorporating rotation-based training (an educational program in which newly hired nurses rotate through different hospital wards before being permanently assigned to one) was launched in April 2008 as part of the education program for newly graduated nurses.

After joining the hospital in April, newly graduated nurses undergo rotation-based training in three wards (surgical, medical, and other departments) before being formally assigned to a unit in November. The rotation consists of three rounds: the first lasting 14 weeks, the second 12 weeks, and the third 10 weeks.

Although previous studies reported that newly graduated nurses undergoing rotation-based training experience anxiety about their clinical practice and difficulties related to moving between multiple departments in a short period [6], the psychological state of these nurses for each rotation phase over a six-year period has yet to be examined. In this study, psychological state refers to the emotional and cognitive reactions experienced by newly graduated nurses during each rotation phase, including anxiety, stress, self-efficacy, job satisfaction, and motivation [4,6].

Since 2009, in addition to the preceptorship system, Fukui University Hospital has assigned two clinical coaches (nurses who provide support and guidance to newly graduated nurses) to each department as part of the education system for newly graduated nurses. Psychological distress among nurses can have a significant impact on nursing practice. Stress and anxiety may lower concentration and judgment, leading to errors in patient care and making it difficult to build trusting relationships with patients. Persistent psychological distress can result in burnout, decreased motivation and job satisfaction, and an increased risk of resignation. It may also affect communication and teamwork within the workplace, causing disruptions in coordinated care. Furthermore, prolonged distress can lead to physical symptoms such as sleep disturbance and headaches, and reduce nurses’ capacity to make sound ethical decisions.

Based on insights from clinical coaching practice, the importance of providing newly graduated nurses with evidence-based and well-reasoned support was identified. Hickey emphasized the importance of developing a structured training program that allows nurse educators to systematically and effectively foster newly graduated nurses, ensuring appropriate staffing levels to maintain the quality of education, and providing sufficient support for both educators and newly graduated nurses to prevent turnover [7-9].

To facilitate more effective guidance for newly graduated nurses, this study investigated their psychological distress during each rotation phase and sought evidence to enhance educational support provided by nurse educators. The present study aimed to identify the timing and nature of psychological distress experienced by newly graduated nurses during rotation-based training and to determine the specific types of support required to enhance their coping and adaptation (Murabayashi et al.) and [9-12].

## Materials and methods

Methods

A quantitative descriptive design was employed to examine the psychological distress experienced by newly graduated nurses during rotation-based training. The investigation was conducted from April 2008 to December 2013. A total of 301 newly graduated full-time nurses who entered the rotation-based training program at Fukui Hospital between fiscal years 2008 and 2013 were eligible for the study, and all were invited to participate (census sampling). Among them, 299 provided informed consent, and 230 (76.9%) responded to the survey and were included in the analysis. Two nurses who did not provide informed consent were excluded from the analysis.

Data were collected using a self-administered, self-developed questionnaire. The questionnaire was created by the authors because no validated tool was available in Japan to assess psychological distress among newly graduated nurses in rotation-based training. It consisted of multiple-choice items on the timing and nature of psychological distress, as well as open-ended questions about specific difficulties experienced during training. The content validity of the questionnaire was reviewed by three nursing education experts, and a pilot test was conducted before formal administration.

The survey was administered once per participant, one week after completion of each rotation round. Data were collected for the first and second rotations; the third rotation was excluded because of incomplete responses.

Quantitative and qualitative data were analyzed separately. Descriptive quantitative analysis was conducted using Microsoft Excel (Microsoft Corp., Redmond, WA), calculating frequencies and percentages. For qualitative data, the free-text responses were analyzed using text-mining techniques to explore linguistic patterns in participants’ descriptions of psychological distress. Specifically, word frequency and co-occurrence analyses were performed using KH Coder (version 3; developed by Koichi Higuchi, Ritsumeikan University, Kyoto, Japan) to identify frequently occurring terms and their interrelationships. No inferential statistical analyses were performed in this study.

Data were collected using a self-administered, self-developed questionnaire designed to assess psychological distress experienced during each rotation phase. It included closed-ended (two-point) items and open-ended questions that allowed participants to describe their experiences in detail. The questionnaire was reviewed by three nursing education experts to confirm content validity, and a pilot test was conducted before the main survey. The questionnaire items are presented in Appendix 1.

Ethical considerations

This study was approved by the Ethics Committee of the University of Fukui Hospital (Approval No. H1-01), and participants were provided with both verbal and written explanations of the purpose of the study and its privacy policy. They were informed that their participation was voluntary, that it would not affect their job responsibilities, and that the data would be aggregated for statistical analysis, with no personal information included in the results. Written informed consent was obtained before participation in the study. An anonymous, self-administered questionnaire was used for this study. 

## Results

Quantitative findings

Regarding the presence of psychological distress, among the 299 newly graduated nurses who consented to participate, 230 (76.9%) reported experiencing psychological distress during the first rotation, and 210 (70.2%) during the second rotation. The period identified as the most psychologically distressing was the first rotation in July, reported by 177 nurses (61.03%). Table 1 summarizes the frequency and percentage of participants who reported experiencing psychological distress.

**Table 1:** Psychological distress reported during rotation training (N=299)

Rotation period	Experienced psychological distress (n)	Percentage (%)	No psychological distress (n)	Percentage (%)
First rotation	230	76.9	69	23.1
Second rotation	210	70.2	89	29.8

**Figure 1:**
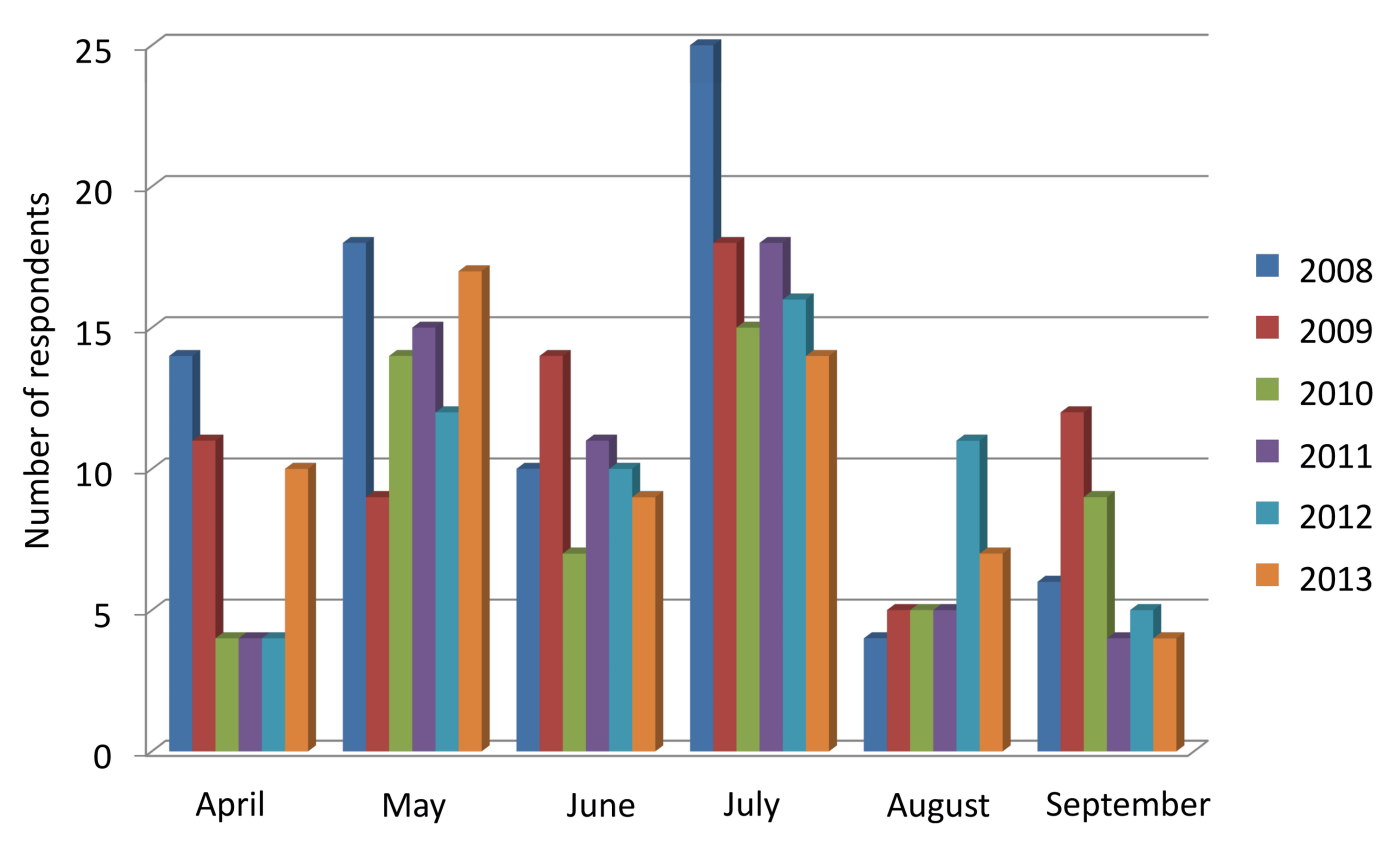
Timing of psychological distress experienced during rotation-based training Newly graduated nurses at Fukui University Hospital (N=299, 2008–2013)

Regarding the timing of psychological distress (Figure 1), July was most frequently reported during the first rotation, followed by May during the second rotation. Specifically, in the first rotation, 230 out of 299 (76.9%) participants reported experiencing distress in July, while in the second rotation, 146 (69.5%) participants identified May as the period of greatest distress.

In terms of the content of the distress, the word frequency analysis of free-text responses revealed differences in frequently appearing words across the different rotation rounds.

According to the text-mining analysis, differences were observed in the frequency of words appearing in each rotation round. In the first rotation round, the most frequently appearing words were, in descending order, “work” (45 times), “myself” (39 times), “too much” (35 times), “painful” (24 times), and “not + able” (19 times).

**Figure 2:**
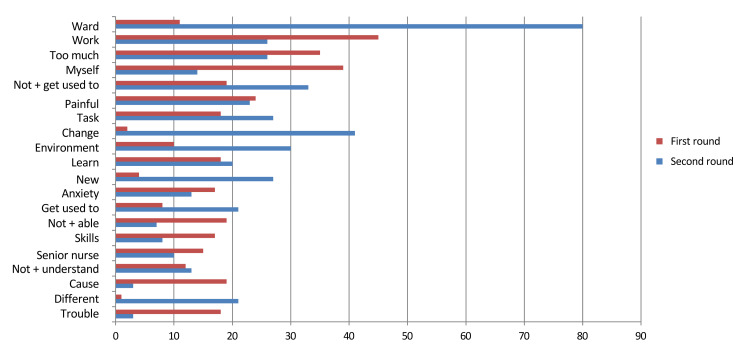
Text mining: Word frequency analysis chart Newly graduated nurses at Fukui University Hospital (first round: N＝230, second round: N＝210, 2008–2013)

In the second rotation round, the most frequently appearing words were “ward” (80 times), “change” (41 times), “not + get used to” (33 times), “environment” (30 times), and “new” (27 times) (Figure 2).

Representative statements from participants illustrate the context of these frequently occurring words. During the first rotation, nurses reported: “I felt distressed because I couldn’t handle my work well.”, “It was painful not being able to remember the tasks.”, “I felt depressed and didn’t want to go to work.”, “It was difficult when I gradually realized how demanding the work was.”, and “I was desperate to learn the flow of work.”

These statements reflect the emotional strain, sense of inadequacy, and difficulty adapting to the clinical environment that characterized the early stage of employment.

**Figure 3:**
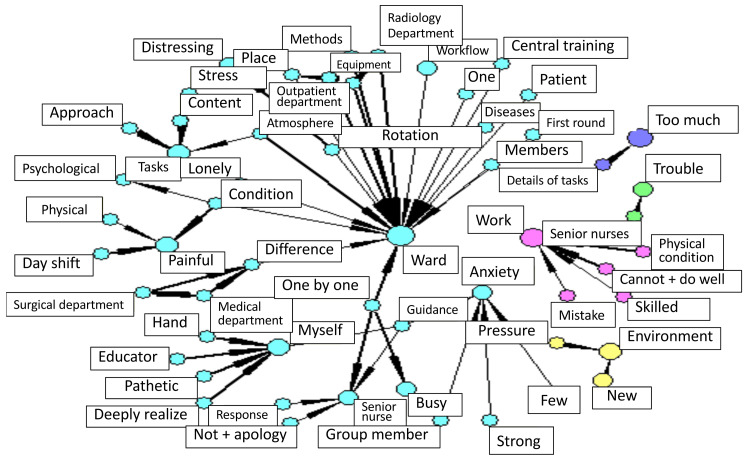
Co-occurrence analysis: Word network map based on six years of data Newly graduated nurses at Fukui University Hospital (N=299, 2008–2013)

This text-mining analysis was conducted for the first and second rotation rounds only; word frequencies for other rotations were not analyzed. In the co-occurrence analysis, the most frequent word “ward” was strongly associated with “members,” “diseases,” “rotation,” “equipment,” “methods,” “atmosphere,” and “differences.” The next most frequent word “work” frequently co-occurred with the expressions “cannot + do well,” “mistake,” “skilled,” and “physical condition (Figure 3).

Representative statements from participants illustrate these relationships. For example: (1) “After being transferred to a new ward, I didn’t even know where the stethoscopes were or how to use the bath facilities, and I had to ask senior nurses each time. It was difficult to speak to them when they were busy.” (2) “When I had just moved to a new ward, I was in an unfamiliar environment and felt mentally exhausted.” (3) “At the beginning of a new rotation, the ward atmosphere and routines were completely different, and I struggled to get used to them.” (4) “It was hard until I became familiar with the atmosphere and relationships within the new ward.” (5) “The rules and workflow differed from the previous ward, and I needed time to adjust.” (6) “Even though I wasn’t used to the ward yet, I sometimes had to handle patients on my own, which made me feel anxious.”

These statements demonstrate that nurses experienced distress related to changes in work environment, interpersonal relationships, and differences in ward culture and routines. The frequent co-occurrence of “ward” with words such as “members,” “methods,” and “atmosphere” reflects the difficulty of adapting to new working conditions, while the linkage between “work” and “mistake” or “cannot + do well” indicates anxiety and loss of confidence in performing nursing tasks.

## Discussion

In this survey, the period when psychological distress was most frequently reported among newly graduated nurses was July, which corresponds to the third month after employment. According to Takeuchi and Sugiyama [13], self-efficacy tends to decline around three months after employment, increasing the intention to resign, with no improvement observed in mental health status. Similarly, Mizuta [14] reported that mental health deteriorates most severely at three months after employment, with approximately 30% of newly graduated nurses exhibiting moderate or higher levels of depressive tendency.

Furthermore, July often coincides with the timing of the first departmental rotation in many medical institutions, which increases stress due to environmental changes. Reality shock and difficulties in workplace adaptation associated with departmental transfers have also been identified as factors that exacerbate the psychological burden of newly graduated nurses [15].

Focusing on the content, during the first rotation period, many nurses expressed distress related to being unable to perform their duties as expected. For example, one nurse stated, “There were so many things I didn’t understand at work that I began to hate myself for being unable to do anything.” This suggests that the nurses were struggling with the complexity and volume of their duties, as well as with their perceived lack of competence.

Tanaka [16] identified “insufficient practical ability in acute-care hospitals” and “negative self-perception of one’s actions” as major challenges faced by newly graduated nurses, clarifying that anxiety about job performance and self-denial are key factors contributing to psychological distress. Moreover, Kobayashi and Nakamura [17] reported that stress responses such as “a sense of incompetence” and “a feeling of excessive pressure” remained at persistently high levels during the first six months after employment. These findings support the conclusion that the complexity of nursing tasks and decreased self-efficacy are central factors in psychological distress.

It is considered that the nurses became acutely aware of their lack of experience and faced a gap between their expectations and reality. During the first one to two months after employment, it is essential to support newly graduated nurses so that they can apply the knowledge and skills acquired during basic nursing education to clinical practice.

During the second rotation period, many nurses reported distress associated with changes in the ward environment. For example, one nurse commented, “I couldn’t get used to the new environment, and I was confused because the procedures were completely different from those in my previous ward.” Even if relationships with senior nurses, physicians, and patients had already been established, they must be rebuilt from the beginning with every rotation. Therefore, during the transitional phase following the first rotation, support should be provided to help newly graduated nurses form new interpersonal relationships.

Clinical coaches with more than five years of nursing experience and extensive clinical knowledge can serve as effective bridges between newly graduated nurses and other staff members. Furthermore, even when the arrangement of materials and details of nursing procedures differ among wards, information exchange and sharing among clinical coaches can promote standardization and correction of inconsistencies throughout the hospital, thereby contributing to a more unified working environment. Continued efforts are required to alleviate psychological distress among newly graduated nurses at each stage of the rotation program.

Word frequency analysis across rotation rounds

First rotation round: Frequently appearing words included “work,” “self,” “many,” “hard,” and “cannot.” At this stage, newly graduated nurses appeared to experience considerable anxiety and stress related to their perceived lack of competence and the large volume of tasks. This phenomenon can be interpreted as a form of “reality shock.”

Murabayashi et al. (The practice and outcomes of rotation-based training for newly graduated nurses; Proceedings of the 57th Annual Meeting of the Japanese Association of Rural Medicine; 2008) reported that rotation training in clinical wards helped alleviate newly graduated nurses’ anxiety and mitigate reality shock, emphasizing the importance of psychological support in the early stages of employment. Such early psychological burdens highlight the necessity of incorporating mental health support systems in the design of rotation training programs.

Second rotation round: Frequently appearing words included “ward,” “change,” “not get used to,” “environment,” and “new.” This indicates that nurses faced challenges in adapting to new environments and changes in their assigned wards.

Mizuguchi et al. [10] noted that rotation training enables nurses to compensate for differences in clinical experience across departments, thereby fostering adaptability to environmental changes. Similarly, Oya et al. [11] identified four categories of effects of rotation training: (1) Acquisition of nursing skills, (2) factors facilitating learning such as supervisory systems, (3) psychological impact on newly graduated nurses, and (4) effects on wards and instructors.

These findings support the importance of designing rotation programs that address adaptation difficulties and anxiety, as reflected by words such as “not get used to,” “change,” and “environment.”

Insights from the co-occurrence analysis of newly graduated nurses’ rotation training experiences

The co-occurrence analysis of newly graduated nurses’ rotation training experiences revealed that the frequently appearing word “ward” was strongly associated with “members,” “diseases,” “workflow,” “equipment,” “methods,” “atmosphere,” and “differences.”

This suggests that variations in the environment and work style among wards greatly influence the learning experiences of newly graduated nurses. In addition, words co-occurring with “work,” such as “cannot,” “mistake,” “skillful,” and “physical condition,” indicate that newly graduated nurses were highly concerned about their skill development, potential mistakes, and physical well-being.

Influence of ward differences on learning

Rotation training enables nurses to broaden their range of clinical skills through experiences in different wards. By directly experiencing variations in ward “atmosphere” and “methods,” nurses develop flexibility and adaptability. A practical report from Hamamatsu University Hospital highlighted that rotation training contributed to enhancing organizational understanding and restructuring educational systems, particularly for newly graduated nurses who lacked sufficient clinical experience during the COVID-19 pandemic.

Technical skill acquisition and psychological burden

The frequent co-occurrence of words such as “mistake” and “cannot” with “work” reflects reality shock and decreased self-efficacy. Previous studies have shown that rotation training helps alleviate reality shock and promotes mental stability among newly graduated nurses (Murabayashi et al.) and [10,12]. These findings support the interpretation that such training serves not only as a technical learning opportunity but also as a form of psychological support.

Educational significance and workplace culture change

Rotation training is not merely a venue for acquiring skills but also contributes to cultivating a workplace culture in which the entire team supports the development of new nurses. Reports have indicated that such training enhances “a sense of solidarity” and leads to “changes in attitudes toward education.”

This aligns with the frequent co-occurrence of the words “members” and “atmosphere,” suggesting that team relationships significantly influence the growth of newly graduated nurses.

Limitations and future directions

This study has several limitations. The participants were limited to newly graduated nurses working at specific hospitals or facilities, which makes it difficult to generalize the findings to other institutions or regions. In addition, the survey period was restricted, and the results may have been influenced by seasonal factors or differences in educational systems across academic years. Furthermore, psychological distress may not be attributable solely to rotation; rather, it could also be affected by various factors such as individual personality, living environment, and relationships with senior staff [18].

Methodologically, the use of text-mining analysis and descriptive data analysis presents inherent limitations. Text mining, while useful for exploring patterns in large volumes of qualitative data, may fail to capture contextual depth, emotional tone, and subtle meaning in participants’ narratives. Descriptive quantitative analysis can illustrate only basic distributions and tendencies, without assessing statistical significance or causal relationships.

Future research should focus on developing and validating a more robust measurement tool to assess psychological distress among newly graduated nurses in rotation-based training. In addition, longitudinal or mixed-methods studies are needed to examine how distress changes over time and how targeted educational or organizational interventions might alleviate it and improve retention.

## Conclusions

This study identified that newly graduated nurses experienced the highest levels of psychological distress around the third month of employment, corresponding to the first departmental rotation. This phase represents a critical adjustment period involving increased clinical responsibility, exposure to new interpersonal relationships, and adaptation to different ward cultures. The analysis revealed that the nature of psychological distress differed by rotation phase: early-phase distress was mainly related to self-efficacy and perceived incompetence, whereas later-phase distress reflected stress from adapting to new organizational norms and expectations. These findings demonstrate that distress among newly graduated nurses is dynamic and context-dependent, even within a structured rotation-based program.

Based on these results, institutions implementing rotation-based training should provide phase-specific support to help nurses cope effectively with transition-related stress. Strategies may include pre-rotation orientation, reflective debriefing sessions, and consistent mentorship across departments. Furthermore, the presence of trained clinical coaches who can facilitate communication, ensure educational continuity, and foster a psychologically safe environment is essential. Future research should develop validated instruments to measure psychological distress during rotational training and evaluate the impact of targeted interventions on nurse adaptation and retention. By integrating such evidence-based support systems, healthcare institutions can enhance the well-being and professional development of newly graduated nurses.

## Appendices

**Table 2:** Questionnaire at the end of each rotation round in the comprehensive nursing competency development program for newly graduated nurses Data were collected using a self-administered, self-developed questionnaire designed to assess psychological distress experienced during each rotation phase. It included both closed-ended (two-point) and open-ended items, reviewed by three nursing education experts to ensure content validity and pilot-tested before data collection.

Question	Response Options
1. Please evaluate the comprehensive nursing competency development program for newly graduated nurses at our hospital.	Satisfied / Somewhat satisfied / Somewhat dissatisfied / Dissatisfied
	If you answered “Somewhat dissatisfied” or “Dissatisfied,” please describe the reasons.
2. Please evaluate the overall guidance provided in each ward.	Satisfied / Somewhat satisfied / Somewhat dissatisfied / Dissatisfied
	If you answered “Somewhat dissatisfied” or “Dissatisfied,” please describe the reasons.
3. To what extent did you achieve the goals of the first round of the comprehensive nursing competency development program?	Mostly achieved / Largely achieved / Barely achieved / Not achieved
4. Please evaluate the length of the rotation period (14 weeks).	A little long / Appropriate / Somewhat short / Short
5. Please evaluate rotating with the same group members.	Satisfied / Somewhat satisfied / Somewhat dissatisfied / Dissatisfied
	If you answered “Somewhat dissatisfied” or “Dissatisfied,” please describe the reasons.
6. Did you experience any periods of psychological distress?	Yes / No
	If yes, around which month(s)?
	Please describe the content of the distress.
	How did you cope with it and overcome the situation?
7. Would you recommend this training program to your juniors or acquaintances?	Yes / No
	If you answered “No,” please describe the reasons.
8. Please describe the advantages and disadvantages of this training program for you.	Advantages: _______
	Disadvantages: _______
9. Please feel free to write any additional comments or opinions regarding the program.	Free description
